# The Interrelationship between Obesity and Race in Breast Cancer Prognosis: A Prospective Cohort Study

**DOI:** 10.21203/rs.3.rs-3338366/v1

**Published:** 2023-09-25

**Authors:** Emma Armstrong Schindler, Cristiane Takita, Fernando Collado-Mesa, Isildinha M. Reis, Wei Zhao, George R. Yang, Laura G. Acosta, Jennifer J. Hu

**Affiliations:** University of Miami Miller School of Medicine: University of Miami School of Medicine

**Keywords:** Breast cancer, race/ethnicity, obesity, tumor stage, prognosis

## Abstract

**Purpose:**

Obesity is associated with an increased breast cancer risk in postmenopausal women and may contribute to worse outcomes. Black women experience higher obesity and breast cancer mortality rates than non-Black women. We examined associations between race, obesity, and clinical tumor stage with breast cancer prognosis.

**Methods:**

We conducted a prospective cohort study in 1,110 breast cancer patients, using univariable and multivariable Cox regression analyses to evaluate the effects of obesity, race/ethnicity, and clinical tumor stage on progression-free and overall survival (PFS and OS).

**Results:**

22% of participants were Black, 64% were Hispanic White, and 14% were non-Hispanic White or another race. 39% of participants were obese (body mass index [BMI] ≥ 30 kg/m^2^). In univariable analyses, tumor stage III-IV was associated with worse PFS and OS compared to tumor stage 0-II (hazard ratio [HR] = 4.68, 95% confidence interval [CI] = 3.52–6.22 for PFS and HR = 5.92, 95% CI = 4.00-8.77 for OS). Multivariable analysis revealed an association between Black race and worse PFS in obese (HR = 2.19, 95% CI = 1.06–4.51) and non-obese (HR = 2.11, 95% CI = 1.05–4.21) women with tumors staged 0-II. Obesity alone was not associated with worse PFS or OS.

**Conclusion:**

Results suggest a complex interrelationship between obesity and race in breast cancer prognosis. The association between Black race and worse PFS in tumor stages 0-II underscores the importance of early intervention in this group. Future studies are warranted to evaluate whether alternative measures of body composition and biomarkers are better prognostic indicators than BMI among Black breast cancer survivors.

## INTRODUCTION

The increasing prevalence of obesity in the United States presents a public health challenge. About 42.4% of American adults are obese and non-Hispanic Black adults are disproportionately affected.[1] Obesity is a risk factor associated with multiple health consequences, including diabetes, hypertension, dyslipidemia, stroke, and all-cause mortality.[2] Importantly, accumulating evidence suggests that obesity is linked to breast cancer incidence, recurrence, and worse clinical outcomes.[3-6]

Women who are obese are more likely to present at a later stage of the disease, with larger tumors and more positive lymph nodes at the time of diagnosis.[4, 7, 8] Additionally, obesity is associated with more treatment complications and reduced efficacy of chemotherapy and hormone therapy, contributing to higher rates of locoregional recurrence compared to non-obese women.[5] Obesity is linked to an increased risk of developing a second primary cancer, particularly of the contralateral breast, endometrium, and colon,[3, 4, 9] and a greater risk of distant metastases at ten-year follow-up.[7] Finally, obesity is associated with worse overall and disease-free survival.[3, 5, 8, 10, 11]

Multiple mechanisms underly the association between obesity and breast cancer outcomes. Increased aromatase activity in adipose tissue raises circulating estrogen levels. Estrogen has a proliferative effect on breast tissue, contributing to incidence and recurrence.[5] Second, adipose tissue is highly metabolically active.[3, 5] Production of proinflammatory cytokines tumor necrosis factor alpha (TNF-alpha) and interleukin six (IL-6) in adipose may contribute to breast cancer pathogenesis.[5] The adipokine leptin, which regulates appetite and energy balance and increases in proportion to body mass index (BMI) is also thought to be implicated in cancer progression and metastasis.[3, 12] High levels of leptin have been shown to promote tumor cell migration and invasion, induce epithelial-to-mesenchymal transition, stimulate angiogenesis, and promote breast cancer stem cell survival.[3]

The prevalence of obesity varies by race and breast cancer prognosis.[13] Although the risk of developing breast cancer is similar in Black and White women, Black women are more likely to die from breast cancer.[13] Racial disparities in prognosis are thought to be driven by multiple biological and non-biological factors.[13] Low socioeconomic status and other social factors experienced by Black women may limit access to healthcare and cause delays in screening, detection, and treatment.[13-15] The higher prevalence of comorbid conditions, including obesity, diabetes, hypertension, and cardiovascular and respiratory disease, among Black women is also hypothesized to contribute to worse clinical outcomes.[5, 13, 14] In addition, the incidence of triple-negative breast cancer (TNBC), an aggressive and treatment-resistant subtype, is also higher among Black women.[14] Biological factors underlying racial differences in outcomes include differences in the tumor microenvironment, gene expression, tumor suppressors, and genetic susceptibility loci.[13, 16]

Multiple studies have identified differences in the expression of cancer-associated genes in Black women compared to White women.[17-19] Because gene expression can be altered by environmental and/or lifestyle factors, epigenetic influences may mediate the link between non-biological factors such as race or obesity, and the biological factors associated with worse breast cancer prognosis.

There is accumulating evidence suggesting that the relationship between obesity, race, and clinical tumor stage in breast cancer prognosis is complex.[16, 20-26] The current study aims to build upon our previous work by evaluating the characteristics associated with progression-free and overall survival in the same racially and ethnically diverse cohort of breast cancer patients.[22] We assess the interrelationship among BMI, race, and clinical tumor stage in breast cancer prognosis to improve patient counseling and guide the development of targeted interventions for high-risk groups.

## METHODS

### Study population

We evaluated 1,115 post-surgical breast cancer patients scheduled to receive adjuvant radiation therapy (RT) at the Sylvester Comprehensive Cancer Center (SCCC) and Jackson Memorial Hospital (JMH) in Miami, Florida, between 2008 and 2014. Participants were recruited for a case-control study and/or a study assessing RT-induced skin toxicity to the intact breast. Each patient completed a self-administered questionnaire with (1) demographic information, (2) self-reported race and ethnicity, (3) self-reported height and weight, and (4) assessment of breast cancer risk factors (including family history, presence of comorbidities, and smoking status). Clinical and pathological tumor characteristics were obtained from pathology reports and medical records. Informed consent was obtained from all participants at the time of enrollment. This study was approved by the Institutional Review Board at the University of Miami and Jackson Memorial Hospital.

Inclusion criteria included female patients aged 18 or older who were diagnosed with breast carcinoma stages 0-IV (American Joint Committee on Cancer), were scheduled to receive treatments at SCCC or JMH between 2008 and 2014 and were able and willing to provide informed consent. Exclusion criteria included patients who were aged less than 18, received prior radiation to the currently treated breast or chest wall, were undergoing concurrent chemotherapy, or were unable to provide written consent. We had a final sample size of 1,110 following the exclusion of participants who were lost to follow-up or had missing information.

### Assessment of patient and clinical variables

We evaluated race (non-Black or Black), obesity status (obese or non-obese), age at diagnosis (< 60 years or ≥ 60 years), and smoking status (never, former, or current) as patient covariates. Former smoking was defined as having smoked 100 or more lifetime cigarettes and current smoking was defined as active smoking. Clinical variables, including estrogen receptor (ER), progesterone receptor (PR), human epidermal growth factor receptor 2 (HER2), and triple-negative (TN) status, as well as clinical tumor stage (0-II or III-IV), were determined using medical records. The clinical tumor stage was based on the American Joint Committee on Cancer staging scheme.[27] Body mass index (BMI) was calculated using the National Institute of Health (NIH) conversion formula using self-reported height and weight at the time of enrollment. In the current study, BMI < 30 was considered non-obese and BMI ≥ 30 was considered obese.

### Assessment of progression-free survival and overall survival

Participants were followed for up to 13 years through a review of the medical records, with the evaluation completed as of July 31, 2021. Progression-free survival (PFS) was defined as the time elapsed from diagnosis to the earliest date of disease progression (second primary, recurrence, metastasis, or death). Overall survival (OS) was defined as the time elapsed from diagnosis to death. Event-free patients were censored at the date of the last follow-up.

## Statistical Analysis

Descriptive statistics (Number, percent) are presented for patient and clinical characteristics stratified by obesity status. The bivariate association of obesity status and patient and clinical characteristics was assessed by a Chi-square test for categorical variables. PFS was defined as the time from diagnosis to recurrence, metastasis, secondary breast cancer, death, or last follow-up, whichever occurred first. OS was defined as the time from diagnosis to death or last follow-up. Event-free patients were censored at the date of the last follow-up. Selected covariates associated with obesity or with PFS or OS were included in the multivariable Cox regression model based on univariable analysis and literature review. PFS and OS were estimated by the Kaplan-Meier method and the associations with race and obesity were assessed by a log-rank test. Univariable Cox proportional hazard analysis was used for potential covariables on time-to-event outcomes of PFS and OS. Multivariable Cox proportional hazard analysis was used to assess the association between pretreatment obesity and race category with PFS and OS adjusted for selected covariables. Results were reported as hazard ratios (HR) with 95% confidence intervals (95% CI). Statistical significance was set at a threshold of *P* < 0.05. The heterogeneity of obesity effect by race and by tumor stage was assessed in multivariable analyses stratified by clinical tumor stage (0-II and III-IV). Data analysis was conducted using SAS (version 9.4, Cary, NC).

## RESULTS

### Patient and clinical characteristics

The study population included 1,110 breast cancer patients, 918 (82.7%) of whom were progression-free by the last follow-up and 192 (17.3%) of whom experienced disease progression (including 105 deaths, seven second-primary cancers, 48 breast cancer recurrences, and 123 metastases). 75 women (6.8%) experienced two or more outcomes. The mean age at diagnosis was 54.7 years (range: 24.5–85.0). 30.5% of participants were ≥ 60 years old at diagnosis and 69.5% were < 60 years old. Most of our sample self-identified as Hispanic White (64.1%), 21.7% of participants identified as Black, and 14.1% identified as Non-Hispanic White or another race/ethnicity. Non-Hispanic White and Other categories were combined due to their similar progression-free and overall survival. 76.4% of participants had stage 0-II disease upon enrollment and 23.6% of participants had stage III-IV disease.

38.7% of participants were obese, defined as BMI ≥ 30 kg/m^2^. A significantly higher proportion of obese participants were ≥ 60 years old at diagnosis (43.2% vs. 36.8% <60 years old; p = 0.044), Black (52.3% vs. 35.0% non-Black; p < 0.001), never smokers (40.4% vs. 39.6% for former smokers vs. 23.7% for current smokers) and had ≥ 2 comorbid conditions (52.4% vs. 44.2% for one comorbidity vs. 26.8% for no comorbidities; p < 0.001). Additional patient and tumor characteristics are summarized in Table 1.

### Univariable Cox regression analysis of progression-free survival

Univariable analysis in Table 2 reveals a significantly worse PFS for Black (HR: 1.63, 95% CI: 1.20–2.22) compared to non-Black race and for Hispanic White (HR: 2.36, 95% CI: 1.30–4.27) or Black (HR: 3.44, 95% CI: 1.85–6.40) race/ethnicity, compared to non-Hispanic White/Other. There was a significantly worse PFS for clinical tumor stages III-IV (HR: 4.68, 95% CI: 3.52–6.22) compared to clinical tumor stages 0-II, and worse PFS for tumors that were ER-negative (HR: 1.83, 95% CI: 1.36–2.45), PR-negative (HR: 1.83, 95% CI: 1.38–2.43), HER2-positive (HR: 1.46, 95% CI: 1.04–2.07), or triple-negative (HR: 1.88, 95% CI: 1.35–2.63).

### Univariable Cox regression analysis of overall survival

Univariable analysis in Table 3 shows a significantly worse OS for Black (HR: 1.63, 95% CI: 1.07–2.47) compared to non-Black race, for Hispanic White (HR: 2.49, 95% CI: 1.08–5.74) or Black (HR: 3.61, 95% CI 1.51–8.64) race/ethnicity compared to non-Hispanic White/Other, and for former smokers (HR: 1.72, 95% CI: 1.17–2.53) compared to never smokers. Current smoking was associated with worse OS, but this was not statistically significant (HR: 1.86, 95% CI: 1.00-3.47). There was a significantly worse OS for clinical tumor stages III-IV (HR: 5.92, 95% CI: 4.00-8.77) compared to tumor stages 0-II, and worse OS for tumors that were ER-negative (HR: 2.04, 95% CI: 1.38–3.02), PR-negative (HR: 2.33, 95% CI: 1.59–3.43), or triple-negative (HR: 2.38, 95% CI: 1.55–3.66).

### Multivariable Cox regression analysis of progression-free survival and overall survival by clinical tumor stage

For combined race and obesity at clinical tumor stages 0-II (Table 4A), being Black and obese or Black and non-obese was associated with worse PFS (HR: 2.11, 95% CI: 1.05–4.21 for Black-obese and HR: 2.19, 95% CI: 1.06–4.51 for Black-non-obese). Former smoking was associated with worse OS (HR: 2.82, 95% CI: 1.47–5.41). Current smoking was also associated with worse OS (HR: 2.61, 95% CI: 0.95–7.12), though this was not statistically significant. At clinical tumor stages III-IV (Table 4B), only TNBC was significantly associated with worse PFS (HR: 1.63, 95% CI: 1.04–2.55) and OS (HR: 2.70, 95% CI: 1.58–4.61).

## DISCUSSION

This study uses a large racially and ethnically diverse population to evaluate patient and clinical characteristics associated with worse PFS and OS in early (clinical tumor stages 0-II) and advanced (clinical tumor stages III-IV) breast cancer. Race and obesity status were combined in multivariable models to evaluate their joint effects. In the early breast cancer group, obese and non-obese Black women had significantly higher hazards of progression compared to non-Black, obese women. Our results emphasize the importance of race as a prognostic indicator in breast cancer that, when combined with obesity status, may contribute to worse outcomes. We know from prior studies that Black women are more likely to have worse breast cancer prognosis despite a similar risk of developing breast cancer compared to their White counterparts.[13] Reasons for this disparity include racial differences in the tumor microenvironment, gene expression, socioeconomic status, and access to healthcare.

We found a significant association between combined race and obesity with worse PFS in early, but not advanced breast cancer (Table 4A and 4B). Differences in gene expression by race and obesity status may underlie disparities in outcomes; such differences may also vary by tumor stage and subtype. For example, Do et al. observed differential hypomethylation of obesity-associated genes in Black women, which was associated with greater all-cause mortality compared to White women.[20] Xing et al. identified increased expression of *SOS1*, a gene that is activated by a compound secreted from adipocytes, implicated in anti-apoptotic pathways, and has been linked to breast cancer progression and metastasis, in Black women compared to White women, as well as altered expression of its epigenetic regulatory elements.[16] *SOS1* is activated by a compound secreted from adipocytes. Finally, resistin is another gene that may mediate this link, as it is associated with obesity, insulin resistance, and breast cancer risk, and is expressed higher in the tumors of Black women than in White women.[21, 24] Like our findings, Vallega et al. observed increased resistin expression in Black women for tumors that were early-stage and receptor-negative.[21] They did not observe any difference in resistin expression in Stage III tumors in interracial comparisons.[21] Importantly, our observation of worse PFS in stage 0-II disease but not at later stages highlights the importance of early intervention strategies in Black women with breast cancer, due to the higher hazard of progression of early-stage disease compared to non-Black women. Future studies are needed to uncover which molecular pathways are differentially activated by race and obesity status and why these pathways are differentially activated, paving the way for potential therapeutic targets and health policy interventions.

Although previous studies suggest that obesity is independently associated with breast cancer incidence, recurrence, and worse clinical outcomes,[3-6] we did not identify an association of obesity with PFS or OS that was independent of race. It is possible that race is a more substantial driver of outcomes than obesity in this cohort, or that the interaction between race and obesity is a stronger driver of outcomes than obesity alone. Previous works highlight the interaction between race and obesity at the molecular level; epigenetic modulation of multiple tumorigenic molecular pathways in adipocytes has been linked to differences in all-cause mortality, progression, and metastasis in Black women compared to White women.[16, 18-21, 28]

The lack of independent association of obesity with PFS or OS may also be attributed to the limitations of BMI as a measure of obesity. Emerging evidence suggests that BMI is an oversimplified metric, as it does not distinguish between muscle and adipose, nor does it describe patterns of adipose distribution.[29-31] Adipose tissue is nonuniform, and while there is some evidence to suggest that subcutaneous fat provides nutritional reserve in advanced cancer, visceral adipose is pro-inflammatory, with a poor cardiometabolic risk profile that promotes tumor growth.[29] In addition, high muscle mass may be linked to better cancer outcomes, whereas low muscle mass has been associated with recurrence, surgical complications, treatment toxicity, and worse OS.[29] Because BMI does not account for muscle mass, those with higher muscle mass may be misclassified as obese despite a potentially lower risk of progression. Finally, in a study of Black breast cancer survivors, higher waist-to-hip ratio and central adiposity were associated with worse breast cancer-specific and overall survival, whereas BMI was not associated with worse outcomes.[30] The findings in our study may reflect the limitations of BMI as a measure of obesity and future studies are needed to evaluate whether central obesity or higher adiposity are more sensitive prognostic indicators for predicting PFS or OS in Black breast cancer survivors.

**Figure 1.**
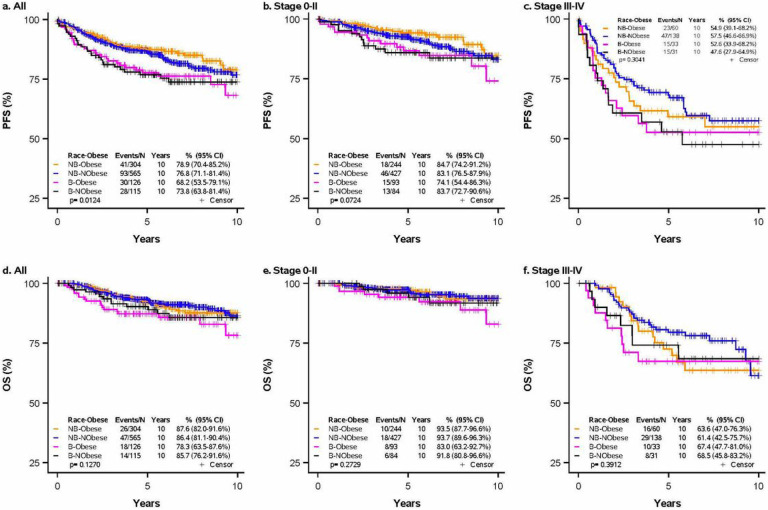
Progress ion-free survival and overall survival by race and obesity Kaplan-Meier curves demonstrating PFS and OS by race and obesity, stratified by clinical tumor stage. **A** PFS in all participants **B** PFS in clinical tumor stage 0-II **C** PFS in clinical tumor stage III-IV **D** OS in all participants **E** OS in clinical tumor stage 0-II **F** OS in clinical tumor stage III-IV. PFS, progression-free survival; OS, overall survival; B, Black race; NB, Non-Black race. Obese was defined as BMI ≥ 30 and non-obese was defined as BMI < 30 (kg/m^2^). *P*-values were determined using a log-rank test; a *p*-value <0.05 was considered statistically significant.

Kaplan Meier survival analyses revealed that non-Black obese women had the lowest hazard of progression among all participants in the tumor stage 0-II group (Fig. 1). Non-Black obese and non-Black non-obese women performed similarly in terms of OS. Accordingly, the non-Black obese group was selected as the reference group in multivariable analyses. Emerging literature describes an ‘obesity paradox’ in which obesity is associated with worse outcomes in early cancer, but is protective at later stages by providing a nutritional reserve to protect against cachexia.[23, 26, 32] The findings in Fig. 1 may reflect a slightly protective effect of obesity. However, we observe this finding in the early-stage group only and not at later stages, which is inconsistent with descriptions of the obesity paradox. Any protective effects of obesity in the advanced group may be obscured by the small sample size attributed to a) the smaller proportion of participants with advanced-stage cancer and b) the smaller proportion of participants with advanced-stage cancer who remain obese despite the associated wasting. Future studies with larger samples are necessary to better characterize the conditions under which obesity may benefit cancer patients.

The stratified multivariable analyses demonstrate an association of TNBC with worse PFS and OS in the advanced breast cancer group. The association with worse outcomes in this cohort is best explained by the aggressiveness of TNBC. TNBC lacks hormone receptor expression and is thus not susceptible to hormonal therapies, leading to worse outcomes.[33, 34] Additionally, TNBC grows faster than other subtypes and is more likely to be diagnosed at a later stage, as evidenced by the higher proportion of patients with TNBC (22.3%) in the advanced breast cancer group compared to the early breast cancer group (13.7%).

Multivariable Cox models revealed a 2.82-fold increased hazard of death in former smokers compared to never smokers in the early-stage breast cancer group (95% CI, 1.47–5.41) (Table 4A). These findings are consistent with the known association of smoking with widespread organ damage, all-cause mortality, and cancer-specific mortality.[35] There was a similar 2.61-fold increased hazard of death associated with current smoking, though this finding was not statistically significant (Table 4A). The lack of significant association of current smoking with PFS or OS in either group or of former smoking with PFS or OS in the advanced-stage group is likely due to the small sample size.

This study has several strengths. First, a prospective study design is appropriate to assess the patient and clinical characteristics associated with PFS and OS. In addition, we utilize a large racially and ethnically diverse cohort that we followed for up to 13 years, enabling us to evaluate inter-group differences in outcomes. Moreover, although many previous studies characterize the patient and clinical characteristics associated with breast cancer risk, few studies focus specifically on outcomes. This work builds upon our previous study in which we used a PRS to evaluate genetic predisposition to obesity in this same racially and ethnically diverse cohort of breast cancer patients using GWAS data.[22] We found high PRS was associated with obesity, Black race, and high CRP levels, all of which have been hypothesized to contribute to breast cancer incidence and worse outcomes. The current study expands upon these findings to explore patient and clinical characteristics associated with prognosis in the same cohort. Having both GWAS and outcome data available for this cohort enables us to conduct future research using genetic prediction models to examine the factors that contribute to breast cancer prognosis.

This study has several limitations. First, the lack of association between race, obesity, and worse PFS or OS in advanced breast cancer could be attributed to the small sample size in this group. Future studies with larger sample sizes are necessary to elucidate potential differences. Second, our use of BMI as a primary outcome may not accurately reflect differences in adiposity. Our decision to use BMI was based on its extensive use in previous studies and the patient data that was available from the study enrollment survey. Third, our patient population was highly enriched for Hispanic White women, (which reflects the racial and ethnic composition of the local population); results may therefore not be generalizable to all populations. Finally, our study did not evaluate variables such as socioeconomic status or access to healthcare, which may also contribute to breast cancer outcomes.

## CONCLUSION

Our findings suggest a complex relationship between obesity and race in breast cancer prognosis. The significant association of combined Black race and obesity status with worse PFS in early-stage breast cancer highlights the importance of targeted early intervention strategies among Black women with breast cancer due to the higher hazard of progression of early-stage disease. An interaction of race and obesity at the molecular level may contribute to the observed differences in PFS; future studies are needed to characterize how environmental and lifestyle factors may alter the expression of cancer-associated genes. Finally, the lack of independent association between BMI alone and PFS or OS may suggest that alternate measures of body composition better illustrate the role of obesity in breast cancer outcomes. Future studies should evaluate whether central obesity and adiposity are more sensitive prognostic indicators than BMI among Black breast cancer survivors.

## Figures and Tables

**Table 1:** Patient and Clinical Characteristics by Obesity Statusa

				Obesity				p- value*
Variable	Category	Total		Non-Obese		Obese		
		N	%	N	%	N	%	
	Total	1,110	100.0	680	61.3	430	38.7	
**Age at diagnosis (years)**	< 60	772	69.5	488	63.2	284	36.8	**0.044**
	≥ 60	338	30.5	192	56.8	146	43.2	
**Race**	Non-Black	869	78.3	565	65.0	304	35.0	**< 0.001**
	Black	241	21.7	115	47.7	126	52.3	
**Ethnicity**	Non-Hispanic	308	27.7	175	56.8	133	43.2	0.06
	Hispanic	802	72.3	505	63.0	297	37.0	
**Race/ethnicity**	NHW/Other^b^	157	14.1	111	70.7	46	29.3	**<0.001**
	HW	712	64.1	454	63.8	258	36.2	
	Black	241	21.7	115	47.7	126	52.3	
**Smoking status**	Never	738	66.5	440	59.6	298	40.4	**0.006**
	Former	275	24.8	166	60.4	109	39.6	
	Current	97	8.7	74	76.3	23	23.7	
**Clinical tumor stage**	0-II	848	76.4	511	60.3	337	39.7	0.218
	III-IV	262	23.6	169	64.5	93	35.5	
**Number of comorbidities** ^c^	0	466	42.0	341	73.2	125	26.8	**< 0.001**
	1	396	35.7	221	55.8	175	44.2	
	2+	248	22.3	118	47.6	130	52.4	
**Family history of breast cancer**	No	730	65.8	449	61.5	281	38.5	0.876
	Yes	372	33.5	227	61.0	145	39.0	
**Family history of any cancer**	No	539	48.6	323	59.9	216	40.1	0.337
	Yes	561	50.5	352	62.7	209	37.3	
**ER status**	Positive	813	73.2	486	59.8	327	40.2	0.077
	Negative	294	26.5	193	65.6	101	34.4	
**PR status**	Positive	697	62.8	415	59.5	282	40.5	0.095
	Negative	407	36.7	263	64.6	144	35.4	
**HER2 status**	Negative	779	70.2	471	60.5	308	39.5	0.349
	Positive	179	16.1	115	64.2	64	35.8	
**Triple-negative status**	Non-TN	903	81.4	547	60.6	356	39.4	0.503
	TN	169	15.2	107	63.3	62	36.7	

NHW, non-Hispanic White; HW, Hispanic White; ER, estrogen receptor; PR, progesterone receptor; HER2, human epidermal growth factor receptor 2; TN, triple negative

a Non-Obese: BMI < 30; Obese: BMI ≥ 30 (kg/m^2^)

b Non-Hispanic White and Other race categories combined due to similar progression and overall survival

* Chi-square test excluding unknowns. A p-value < 0.05 was statistically significant. Significant findings indicated in bold.

c Sum of 11 self-reported comorbid conditions (diabetes, hypertension, sleep apnea, gastroesophageal reflux disease, hyperlipidemia, osteoarthritis, coronary artery disease, heart disease, fatty liver disease, tuberculosis, or other)

**Table 2:** Univariable Cox regression analysis of progression-free survival

Variable	Category	N	Progression				HR (95% CI)
			No		Yes		
			N	%	N	%	
	Total	1,110	918	82.7	192	17.3	
**Age at diagnosis (years)**	< 60	772	640	82.9	132	17.1	Ref
	≥ 60	338	278	82.2	60	17.8	1.11 (0.82, 1.51)
**Race**	Non-Black	869	735	84.6	134	15.4	Ref
	Black	241	183	75.9	58	24.1	**1.63 (1.20, 2.22)**
**Ethnicity**	Non-Hispanic	308	254	82.5	54	17.5	Ref
	Hispanic	802	664	82.8	138	17.2	1.02 (0.75, 1.40)
**Race/ethnicity**	NHW/Other	157	145	92.4	12	7.6	Ref
	HW	712	590	82.9	122	17.1	**2.36 (1.30, 4.27)**
	Black	241	183	75.9	58	24.1	**3.44 (1.85, 6.40)**
**Clinical tumor stage**	0-II	848	756	89.2	92	10.8	Ref
	III-IV	262	162	61.8	100	38.2	**4.68 (3.52, 6.22)**
**Obesity** ^a^	No	680	559	82.2	121	17.8	Ref
	Yes	430	359	83.5	71	16.5	0.94 (0.70, 1.25)
**Smoking status**	Never	738	620	84.0	118	16.0	Ref
	Former	275	218	79.3	57	20.7	1.28 (0.93, 1.76)
	Current	97	80	82.5	17	17.5	1.22 (0.74, 2.04)
**Number of comorbidities** ^b^	0	466	376	80.7	90	19.3	Ref
	1	396	334	84.3	62	15.7	0.76 (0.55, 1.06)
	2+	248	208	83.9	40	16.1	0.82 (0.56, 1.18)
**Family history of breast cancer**	No	730	600	82.2	130	17.8	Ref
	Yes	372	311	83.6	61	16.4	0.86 (0.63, 1.17)
**Family history of any cancer**	No	539	443	82.2	96	17.8	Ref
	Yes	561	466	83.1	95	16.9	0.94 (0.71, 1.25)
**ER status**	Positive	813	692	85.1	121	14.9	Ref
	Negative	294	223	75.9	71	24.1	**1.83 (1.36, 2.45)**
**PR status**	Positive	697	600	86.1	97	13.9	Ref
	Negative	407	312	76.7	95	23.3.	**1.83 (1.38, 2.43)**
**HER2 status**	Negative	779	641	82.3	138	17.7	Ref
	Positive	179	137	76.5	42	23.5	**1.46 (1.04, 2.07)**
**Triple-negative status**	Non-TN	903	761	84.3	142	15.7	Ref
	TN	169	124	73.4	45	26.6	**1.88 (1.35, 2.63)**

NHW, non-Hispanic White; HW, Hispanic White; ER, estrogen receptor; PR, progesterone receptor; HER2, human epidermal growth factor receptor 2; TN, triple negative

Findings in bold indicate significant p-value < 0.05

a Non-Obese: BMI < 30; Obese: BMI ≥ 30 (kg/m^2^)

b Sum of 11 self-reported comorbid conditions (diabetes, hypertension, sleep apnea, gastroesophageal reflux disease, hyperlipidemia, osteoarthritis, coronary artery disease, heart disease, fatty liver disease, tuberculosis, or other)

**Table 3:** Univariable Cox regression analysis of overall survival

Variable	Category	N	Survival				HR (95% CI)
			Alive		Dead		
			N	%	N	%	
	Total	1,110	1,005	90.5	105	9.5	
**Age at diagnosis (years)**	< 60	772	701	90.8	71	9.2	Ref
	≥ 60	338	304	89.9	34	10.1	1.19 (0.79, 1.79)
**Race**	Non-Black	869	796	91.6	73	8.4	Ref
	Black	241	209	86.9	32	13.3	**1.63 (1.07, 2.47)**
**Ethnicity**	Non-Hispanic	308	277	89.9	31	10.1	Ref
	Hispanic	802	728	90.8	74	9.2	0.95 (0.62, 1.44)
**Race/ethnicity**	NHW/Other	157	151	96.2	6	3.8	Ref
	HW	712	645	90.6	67	9.4	**2.49 (1.08, 5.74)**
	Black	241	209	86.7	32	13.3	**3.61 (1.51, 8.64)**
**Clinical tumor stage**	0-II	848	806	95.0	42	5.0	Ref
	III-IV	262	199	76.0	63	24.0	**5.92 (4.00, 8.77)**
**Obesity** ^a^	No	680	619	91.0	61	9.0	Ref
	Yes	430	386	89.8	44	10.2	1.16 (0.79, 1.71)
**Smoking status**	Never	738	681	92.3	57	7.7	Ref
	Former	275	239	86.9	36	13.1	**1.68 (1.11, 2.55)**
	Current	97	85	87.6	12	12.4	1.86 (1.00, 3.47)
**Number of comorbidities** ^b^	0	466	420	90.1	46	9.9	Ref
	1	396	364	91.9	32	8.1	0.79 (0.50, 1.24)
	2+	248	221	89.1	27	10.9	1.11 (0.69, 1.79)
**Family history of breast cancer**	No	730	664	91.0	66	9.0	Ref
	Yes	372	333	89.5	39	10.5	1.11 (0.75, 1.65)
**Family history of any cancer**	No	539	479	88.9	60	11.1	Ref
	Yes	561	516	92.0	45	8.0	0.70 (0.48, 1.04)
**ER status**	Positive	813	750	92.0	63	7.7	Ref
	Negative	294	252	85.7	42	14.3	**2.04 (1.38, 3.02)**
**PR status**	Positive	697	651	93.4	46	6.6	Ref
	Negative	407	348	85.5	59	14.5	**2.33 (1.59, 3.43)**
**HER2 status**	Negative	779	702	90.1	77	9.9	Ref
	Positive	179	158	88.3	21	11.7	1.25 (0.77, 2.03)
**Triple-negative status**	Non-TN	903	830	91.9	73	8.1	Ref
	TN	169	140	82.8	29	17.2	**2.38 (1.55, 3.66)**

NHW, non-Hispanic White; HW, Hispanic White; ER, estrogen receptor; PR, progesterone receptor; HER2, human epidermal growth factor receptor 2; TN, triple negative

Findings in bold indicate significant p-value < 0.05

a Non-Obese: BMI < 30; Obese: BMI ≥ 30 (kg/m^2^)

b Sum of 11 self-reported comorbid conditions (diabetes, hypertension, sleep apnea, gastroesophageal reflux disease, hyperlipidemia, osteoarthritis, coronary artery disease, heart disease, fatty liver disease, tuberculosis, or other)

**Table 4.** Multivariable Cox regression analysis of progression-free survival and overall survival by clinical tumor stage

1. Stage 0-II												
Variable	Category	N	Progression				HR (95% CI)	Vital Status				HR (95% CI)
			No		Yes			Alive		Dead		
			N	%	N	%		N	%	N	%	
	Total	848	756	89.2	92	10.8		806	95.0	42	5.0	

**Age at diagnosis (years)**	<60	573	511	89.2	62	10.8	Ref	546	95.6	27	4.7	Ref
	≥60	275	245	89.1	30	10.9	1.14 (0.73, 1.76)	260	94.5	15	5.5	1.32 (0.70, 2.49)

**Black race and obesity status**	Non-Black obese	244	226	92.6	18	7.4	Ref	234	95.9	10	4.1	Ref
	Non-Black non-obese	427	381	89.2	46	10.8	1.43 (0.83, 2.47)	409	50.7	18	42.9	1.03 (0.47, 2.24)
	Black non-obese	84	71	84.5	13	15.5	**2.19 (1.06, 4.51)**	78	92.9	6	7.1	1.96 (0.71, 5.57)
	Black obese	93	78	83.9	15	16.1	**2.11 (1.05, 4.21)**	85	91.4	8	8.6	2.25 (0.87, 5.79)

**Smoking status**	Never	561	508	90.6	53	9.4	Ref	543	96.8	18	3.2	Ref
	Former	215	184	85.6	31	14.4	1.55 (0.99, 2.43)	196	91.2	19	8.8	**2.82 (1.47, 5.41)** *
	Current	72	64	88.9	8	11.1	1.29 (0.61, 2.74)	67	93.1	5	6.9	2.61 (0.95, 7.12)

**Triple-negative status**	Non-TN	701	629	89.7	72	10.3	Ref	666	95.0	35	5.0	Ref
	TN	111	95	85.6	16	14.4	1.38 (0.79, 2.38)	106	95.5	5	4.5	0.87 (0.34, 2.26)
(B) Stage III-IV												
Variable	Category	N	Progression				HR (95% CI)	Vital Status				HR (95% CI)
			No		Yes			Alive		Dead		
			N	%	N	%	N	%	N	%		
	Total	262	162	61.8	100	38.2		199	76.0	63	24.0	

**Age at diagnosis (years)**	<60	199	129	64.8	70	35.2	Ref	155	77.9	44	22.1	Ref
	≥60	63	33	52.4	30	47.6	1.45 (0.94, 2.23)	44	69.8	19	30.2	1.48 (0.85, 2.57)

**Black race and obesity status**	Non-Black obese	60	37	61.7	23	38.3	Ref	44	73.3	16	26.7	Ref
	Non-Black non-obese	138	91	65.9	47	34.1	0.91 (0.55, 1.50)	109	79.0	29	21.0	0.81 (0.44, 1.50)
	Black non-obese	31	16	9.9	15	15.0	1.42 (0.72, 2.78)	23	74.2	8	25.8	1.04 (0.43, 2.53)
	Black obese	33	18	11.1	15	15.0	1.37 (0.70, 2.67)	23	69.7	10	30.3	1.51 (0.67, 3.40)

**Smoking status**	Never	177	112	63.3	65	36.7	Ref	138	78.0	39	22.0	Ref
	Former	60	34	56.7	26	43.3	1.32 (0.82, 2.12)	43	71.7	17	28.3	1.37 (0.75, 2.51)
	Current	25	16	64.0	9	36.0	0.97 (0.47, 2.01)	18	72.0	7	28.0	1.36 (0.58, 3.18)

**Triple-negative status**	Non-TN	202	132	65.3	70	34.7	Ref	164	81.2	38	18.8	Ref
	TN	58	29	50.0	29	50.0	**1.63 (1.04, 2.55)**	34	58.6	24	41.4	**2.70 (1.58, 4.61)**

Findings in bold indicate significant p-value < 0.05

* Combined current and former smoking yielded significant association with worse OS (HR: 2.77, 95% CI: 1.50-5.13)

## Data Availability

The data sets generated and analyzed during the current study are available from the corresponding author upon reasonable request.

## Abbreviations

BMI: body mass index
CI: confidence interval
ER: estrogen receptor
HR: hazard ratio
HER2: human epidermal growth factor receptor 2
OS: overall survival
PR: progesterone receptor
PFS: progression-free survival
TNBC: triple-negative breast cancer
